# Primary papillary carcinoma of the thyroglossal duct in a 14-year-old female: case report and review of the literature

**DOI:** 10.11604/pamj.2019.32.121.15246

**Published:** 2019-03-14

**Authors:** Amel El Korbi, Jihène Houas, Rachida Bouatay, Khaled Harrathi, Jamel Koubaa

**Affiliations:** 1 ENT Department, Fattouma Bourguiba Hospital of Monastir, University of Monastir, Monastir, Tunisia; 2 Healthcare Research Unit UR12SP41, Tunisia

**Keywords:** Thyroglossal cyst, papillary carcinoma, surgery

## Abstract

Thyroglossal duct carcinoma is a rare pathologic entity. The surgeon's main concern is whether to perform thyroidectomy or not. In this paper, we report another case of thyroglossal papillary duct carcinoma in a 14-old girl suspected preoperatively and confirmed postoperatively on the histological analysis of resected specimen by a Sistrunk procedure. Therapeutic strategy was completed by a total thyroidectomy with radioactive iodine therapy and suppressive levothyroxine therapy. In the absence of clear guidelines, the management of thyroglossal duct carcinoma is depending on the clinical situation and the experience of the team of surgeons.

## Introduction

Thyroglossal duct cysts (TDCs) are the commonest congenital anomaly in the development of the thyroid gland. Classically, it manifests as a swelling in the midline of the neck that moves with deglutition and protrusion of the tongue. The development of a carcinoma in a cyst is rare but well recognized. Brentano *et al*. reported the first description of a thyroglossal duct carcinoma (TDCa) in 1911. It occurs in 0.7-1% of cases and most are undiagnosed preoperatively. TDCa in children is even rarer than in adults, with few cases reported in the English literature [1]. Most of these malignancies share a common histology with those of the main thyroid gland and most of them are papillary carcinoma (75-85%). The relatively small number of patients has always made it difficult to draw any conclusions about their diagnosis and treatment. Although recommendations have been proposed for adults, limited guidance exists regarding management in children.

## Patient and observation

**Figure 1:**
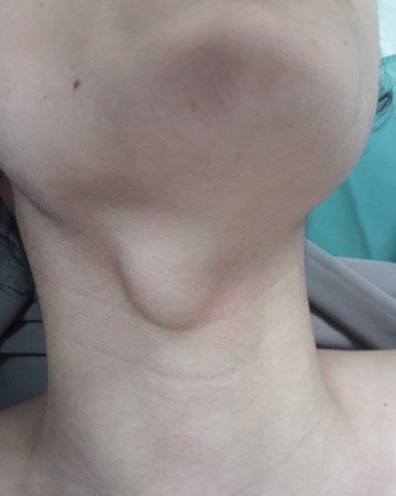
Physical examination revealed a 3cm mass of located in the midline region of the neck covered by a normal skin

**Figure 2:**
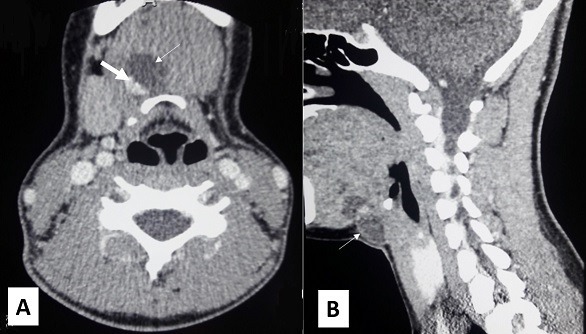
Axial (A) and sagittal (B) with contrast-enhanced computed tomography scan showed a well-circurcumbed cystic lesion attached to the hyoid bone (thin arrow) with a calcified component (thin arrow)

A 14-year-old female patient presented with an 11-month history of neck swelling. She had no dyspnea, dysphagia or hoarseness. There was no history of prior radiation. Familiar medical history was negative for thyroid gland or neoplastic diseases. She did not have any symptoms of hyper-or hypothyroidism. Physical examination revealed a 3cm mass of located in the midline region of the neck covered by a normal skin (Figure 1). It was mobile with deglutition and protrusion of the tongue. It was painless, not tender, well demarcated and not attached to the overlying skin. The thyroid gland was apparently normal in size and consistence with no clinically significant cervical adenopathy. A neck ultrasound confirmed the diagnosis of a thyroglossal cyst. The cystic mass was well-defined, situated in the left paramedian region at the level of the hyoid bone, with a heterogenous component that showed calcifications. There was no significant cervical lymphadenopathy. The thyroid gland was normal. The patient underwent CT before and after intravenous contrast medium. It revealed a lobulated cystic lesion (2cm × 1.8cm × 3.3cm) with a calcified component (Figure 2). This lesion was attached to the anterior hyoid bone with extension from the base of the tongue and deep tongue musculature. The solid component was heterogeneously and intensely enhanced.

**Figure 3:**
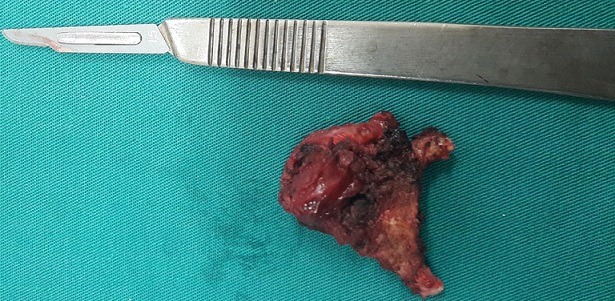
The surgical resected specimen: the cyst attached to the hyoid bone

**Treatment:** A Sistrunk surgical procedure was then performed. Peroperatively, a cystic mass was noted close to the hyoid bone; it was adherent to the digastric and to the mylohyoid muscles. The mass was removed along with entire duct from the gland to the level of the foramen caecum and the middle portion of the hyoid bone. Extemporaneous examination of the surgical specimen showed a cystic mass with a smooth external surface with no signs of malignancy (Figure 3). The definitive histological analysis concluded to the diagnosis of a papillary carcinoma of 1cm arising from a TDC that invaded the adjacent muscles. After discussion in our staff, we have decided to complete by a radioactive iodine therapy because of the microscopic invasion of the adjacent muscles. Therefore, a total thyroidectomy without a neck dissection, were performed with no postoperative complications. Microscopic analysis of the gland was negative for malignancy. The patient underwent a radioactive iodine therapy with suppressive levothyroxine therapy.

**Outcome and follow-up:** Radioactive iodine total body scans have revealed no remnant thyroid tissue. The patient's serum thyroglobulin levels were undetectable 12 months after follow-up.

## Discussion

Thyroglossal duct cysts (TDCs) are the most common anomaly of the cervical region in childhood accounting for 70% of all congenital neck lesions [2]. They originate from the persistence of the thyroglossal duct epithelium in the route of the descent of the thyroid gland to the anterior lower neck region. The malignant transformation of the TDCs is uncommon. It is diagnosed in approximately 0.7% to 1% of thyroglossal duct remnants [3]. Despite TDCs being very common in children and adolescents, carcinomas of the TDCs are rarely present in this age group. This explains why, in the pediatric population, all studies of thyroglossal duct carcinomas (TDCa) are isolated case reports. Reviewing the English literature from 2003 to 2017 we have retrieved thirty-three cases of childhood TDCa. Preoperative evaluation of patients with suspected TDCa varies in clinical practice. It includes physical examination, imaging (neck ultrasonography, neck CT, magnetic resonance imaging) and fine-needle aspiration cytology [4]. The clinical presentation is not specific and therefore, the diagnosis of malignancy was almost made at the time of histological examination. The malignancy should be suspected if the cyst is hard, fixed, irregular and when enlarged lymph nodes are present [5]. In our case, the presence of calcifications in the cyst was suggestive of malignant lesion that is why an extemporaneous examination of surgical specimen was performed. The histologic findings of TDCa are most commonly papillary carcinomas (75-80%) [6]. Other thyroid tumors such as follicular, Hürthle cell, mixed follicular-papillary, squamous cell and other types of carcinomas have been reported [7, 8]. Squamous cell carcinoma is hypothesized to originate from cyst lining and tends to have a worse prognosis [9, 10]. Medullary variants have not been reported because they arise from parafollicular cells embryologically unrelated to the thyroid In 2014. Pfeiffer reported that 21 out of the 26 cases of pediatric patients with TDCa reported in English literature were papillary carcinomas [1]. The type of the tumor in our case was a papillary thyroid carcinoma. In the literature, several case reports have documented these rare lesions, and some authors has suggested that on ultrasound examination, the cancer will appear along the duct wall, as a mural lesion and may have microcalcifications. The sonographic evaluation of the main thyroid gland may confirm an eventual multifocal cancer [10, 11]. Computed tomography (CT) or magnetic resonance imaging (MRI) are rarely justified during the preoperative assessment of presumed TDCs. However, if performed, a TDC is a well-circumscribed, low-density lesion with a smooth, thin wall. Rim contrast enhancement may also be observed. The malignant component is a peripheral mass within the cyst, as a solid mass along the thyroglossal tract, or as a complex invasive midline mass. Ultrasound, CT, or MRI imaging studies can also be used to evaluate the lateral and central neck for nodal metastases. Owing to its rarity, the management of carcinoma arising in a thyroid duct cyst is less well-defined. The extent of surgical management of TDCa remains controversial. Several suggestions have been made for treatment in adults [11, 12].

In all instances, a Sistrunk procedure should be performed, consisting of excision of the cyst, the middle portion of the body of the hyoid bone, and a core of tissue around the thyroglossal tract at the foramen caecum [13, 14]. The next step in management pertains to the thyroid. The currently prevailing theory is that papillary carcinomas arise from the TDCs de novo and are not metastatic from the thyroid gland [1]. Pfeiffer *et al*. reviewed a treatment of twenty-six of childhood TDCa. Thirteen of the patients underwent a total thyroidectomy but none of them had showed a malignant involvement of the thyroid gland. They concluded that Sistrunk procedure and local lymph node resection is sufficient for adolescent patients with TDCa that show no signs of thyroid involvement on ultrasound or uptake scan. Close follow up with these patients is warranted to monitor for future signs of thyroid involvement [1]. In a retrospective study of 57 cases, Patel *et al*. concluded that the addition of total thyroidectomy to the Sistrunk operation did not have a significant impact on recurrence and survival [15]. However, some authors propose a more radical treatment, which involves local excision of the tumor in addition to total thyroidectomy because of occurrence of multicentric papillary carcinomas throughout the entire thyroid gland [14]. Because of co-existent thyroid malignancy with TDCa, Miccoli *et al*. recommend in addition to Sistrunk procedure, a total thyroidectomy and excision of large regional lymph nodes in treating patients with TDCa. Post-operative radioiodine ablation and suppressive levothyroxine are further steps in treatment [13]. The incidence of cervical lymph node metastasis is lower than that of papillary carcinoma of the thyroid. Therefore, most authors agree that a neck dissection should be performed only in the presence of positive lymph nodes. In our case, Sistrunk procedure was performed initially with a histological confirmation of TDCa. Then the patient underwent total thyroidectomy followed by a radioactive iodine therapy and suppressive levothyroxine therapy. Annual physical examination, cervical ultrasound and an unstimulated thyroglobulin level are required for recurrence surveillance.

## Conclusion

The lack of consensus in the management of childhood is due to the rarity of the disease and the small number of cases managed, even in tertiary referral centers. Considering literature and our observations through this case report, we thought that total thyroidectomy must not systematically performed in children and discussed when there is clinical and radiological criteria of gland invasion.

## Competing interests

The authors declare no competing interests.
